# UHPLC-MS/MS-based plasma untargeted lipidomic analysis in patients with diabetes mellitus combined with hyperuricemia

**DOI:** 10.3389/fmolb.2025.1656458

**Published:** 2025-11-13

**Authors:** Youqiong Xu, Yitao Zhu, Qihui Chen, Xinchao Zhang, Jinxi Fang, Wenchu Xu, Xiaoyang Zhang

**Affiliations:** 1 The Affiliated Fuzhou Center for Disease Control and Prevention of Fujian Medical University, Fuzhou, China; 2 School of Public Health, Fujian Medical University, Fuzhou, China

**Keywords:** diabetes mellitus, hyperuricemia, lipid metabolism, lipidomics, triglycerides, phosphatidylethanolamine

## Abstract

**Objective:**

To compare the differences in lipid metabolites between patients with diabetes mellitus combined with hyperuricemia (DH) and diabetes mellitus (DM) and healthy controls, and to initially reveal the changes in lipid metabolic pathways in DH.

**Methods:**

Using a present study method, 17 patients each diagnosed with diabetes mellitus and diabetes mellitus combined with hyperuric acid among permanent residents aged 18 years and above in Fuzhou City, Fujian Province, China, from June 2019 to July 2020 were selected, matched 1:1 by sex and age, and 17 healthy controls were randomly selected and fasting blood samples were collected. The untargeted lipid histology analysis was performed using ultra performance liquid chromatography-tandem mass spectrometry (UHPLC-MS/MS) based technique, and the Student’s t-test and multiple of difference (FC) were used to initially screen the differential lipid molecules and determine their trends; principal component analysis (PCA) and orthogonal partial least squares discriminant analysis (OPLS-DA) were used to observe the overall distribution of the two groups of samples. Finally, we used Metabo Analyst 5.0 platform to analyze the differential lipid metabolism pathways.

**Results:**

We identified 1,361 lipid molecules across 30 subclasses. Multivariate analyses revealed a significant separation trend among the DH, DM, and NGT groups, confirming distinct lipidomic profiles. A total of 31 significantly altered lipid metabolites were pinpointed in the DH group compared to the NGT controls. Among the most relevant individual metabolites, 13 triglycerides (TGs), such as TG (16:0/18:1/18:2), 10 phosphatidylethanolamines (PEs), e.g., PE (18:0/20:4), and 7 phosphatidylcholines (PCs) including PC (36:1), were significantly upregulated, while one phosphatidylinositol (PI) was downregulated. The collective analysis of these metabolite groups revealed their enrichment in six major metabolic pathways. Crucially, glycerophospholipid metabolism with an impact value of 0.199 and glycerolipid metabolism with an impact value of 0.014 were identified as the most significantly perturbed pathways in DH patients. Furthermore, comparison of DH *versus* DM groups identified 12 differential lipids, which were also predominantly enriched in these same core pathways, underscoring their central role in the pathophysiology of hyperuricemia complicating diabetes.

**Conclusion:**

Patients with combined diabetes mellitus and hyperuricemia have significantly altered lipid metabolites compared to diabetic patients and healthy controls. A total of 31 significantly different lipid molecules were identified, and abnormalities in glycerophospholipid metabolism and glycerolipid metabolism pathways were found in DH patients.

## 1 Introduction

Diabetes mellitus (DM) is a group of chronic metabolic diseases caused by impaired insulin secretion, insulin resistance or both, with hyperglycemia as the main feature (Mozaffarian, 2016). According to the International Diabetes Federation’s (IDF) Diabetes Atlas 2021 (Sun et al., 2022),The global prevalence of diabetes in people aged 20–71 years is approximately 10.5% (536.6 million individuals), an increase of 12.9% from the 9.0% prevalence reported in the 2019 IDF. As the disease progresses, diabetic patients are prone to chronic lesions of the fundus, nerves, kidneys, cardiovascular and other tissues and organs, leading to the decompensation or failure of the patient’s organism, which in turn seriously affects the patient’s life and health (Garberg et al., 2025; Hu et al., 2024).

Blood uric acid is a metabolic product of the breakdown of purine nucleotides in the body, and disorders of purine metabolism and/or decreased uric acid excretion can lead to the development of hyperuricemia, which was originally seen in Europe and North America, but with the development of society, the disease has become prevalent in Eastern countries, and the number of cases is gradually increasing in China. In a recent cross-sectional study conducted in 31 provinces in mainland China, 17.7% of the study participants were diagnosed with hyperuricemia (J et al., 2022). Hyperuricemia, a common comorbidity of diabetes (Hyperuricemia, 2017), is inseparable from diabetes. Studies have shown that the incidence of hyperuricemia is higher in diabetic than in non-diabetic population (Anothaisintawee et al., 2017), and that the risk of diabetes increases by 17% for every 1 mL/dL increase in serum uric acid (Kodama et al., 2009), while elevated uric acid levels in diabetic patients are also closely associated with diabetic complications such as diabetic nephropathy, adverse cardiac events and peripheral vascular disease (Katsiki et al., 2013). Both diabetes mellitus and hyperuricemia are metabolic diseases and are often accompanied by lipid abnormalities in the development of the disease (Borges et al., 2010). It has been found that hyperuricemia can lead to lipid abnormalities (Xie et al., 2021), and at the same time, disorders of lipid metabolism are risk factors for diabetes mellitus (Petrenko et al., 2023). However, conventional clinical and blood biomarkers such as BMI, fasting glucose, HbA1c levels, and other conventional biochemical tests cannot capture all lipid molecules, making it necessary to detect early aspects of lipid disorders associated with the disease. In addition, gene expression profiling is limited in exploring the molecular mechanisms of diabetes combined with hyperuricemia given the environmental and genetic heterogeneity (Suomi et al., 2023). Therefore, new approaches are needed to advance the understanding of the mechanisms underlying the development of diabetes mellitus combined with hyperuricemia.

Metabolomics, a rapidly evolving technology, can provide a comprehensive picture of metabolic disorders throughout the body, and it can also provide a new avenue for identifying new biomarkers (Wang et al., 2020). Lipidomics, a branch of metabolomics, is an effective tool to study the changes in lipid metabolism in organisms and the role of lipid regulation in life activities, allowing the identification of many individual lipids and also the ability to characterize the specific biological role of lipid molecules, which is well suited to characterize the lipid perturbations that precede diabetes and can be used to develop specific disease biomarkers (Knebel et al., 2016). Studies have shown that diabetes can be effectively prevented or delayed by lifestyle and pharmacological interventions (Samocha-Bonet et al., 2018). Previous studies have reported the association between lipid alterations and diabetes (Lu et al., 2019; Drogan et al., 2015) and hyperuricemia (Liu et al., 2020; Liu et al., 2022). Alterations in plasma triglycerides (TGs), diglycerides (DAGs), phosphatidylethanolamines (PEs) and phosphatidylcholine (PC) have been associated with type 2 diabetes in different populations (Lu et al., 2019; Drogan et al., 2015). Significant alterations in plasma lipids such as TGs and PCs were identified in patients with hyperuricemia (Liu et al., 2020). However, most of the available studies measured only baseline lipids in patients with diabetes or hyperuricemia and did not reflect changes in the plasma lipidome in patients with diabetes or diabetes combined with hyperuricemia.

In this study, we used ultra-high liquid chromatography-mass spectrometry (UHPLC-MS/MS) to characterize lipid metabolic profiles in patients with diabetes mellitus and diabetes mellitus combined with hyperuricemia. In addition, we identified differentially expressed lipid molecules and enriched metabolic pathways in patients with diabetes mellitus, diabetes mellitus combined with hyperuricemia, and healthy subjects to elucidate the potential role of lipid molecules in detecting the development of diabetes mellitus, diabetes mellitus combined with hyperuricemia.

## 2 Materials & methods

### 2.1 Study population

Multi-stage proportional stratified whole-group sampling method was used to sample 17 patients each diagnosed with diabetes mellitus, diabetes mellitus combined with hyperuricemia, and healthy controls in Fuzhou City, Fujian Province, selected from 2019 to 2020, based on this present study, according to the 1:1 matched case-control study method. Inclusion criteria were (1) 18 years of age or older; (2) completion of questionnaires and blood collection; (3) signing of informed consent; and (4) meeting the American Diabetes Association’s diagnostic criteria (2018) WHO diagnostic criteria for diabetes (2, 2018), i.e., fasting blood glucose ≥7.0 mmol/L, or random blood glucose >11.0 mmol/L, and meeting in the survey Fasting blood uric acid levels were higher than 420 μmol/L in men and 360 μmol/L in women. exclusion criteria: (1) use of hypoglycemic agents; (2) recent use of drugs affecting uric acid metabolism, such as diuretics, lipid-lowering drugs, aspirin, benzbromarone, and allopurin; (3) gout, primary kidney disease, renal insufficiency, leukemia, and tumors; (4) combination of other psychiatric (4) combination of other psychiatric diseases or low cooperation; (5) pregnant and lactating women. This study was approved by the Ethics Committee of the Fuzhou Center for Disease Control and Prevention (approval number: 2,019,006).

### 2.2 Sample collection and pre-processing

5 mL of fasting morning blood was collected and centrifuged at 3,000 rmp for 10 min at room temperature. 0.2 mL of the upper layer of plasma was divided into 1.5 mL centrifuge tubes, and three equal groups of samples were mixed as quality control samples and stored at −80 °C in the refrigerator. The samples were thawed on ice and vortexed, 100 μL was taken into a 1.5 mL centrifuge tube, 200 μL of 4 °C water was added, 240 μL of pre-cooled methanol was added after mixing, 800 μL of methyl tert-butyl ether (MTBE) was added after mixing, 20 min of sonication in a low temperature water bath, 30 min of standing at room temperature, 14 000 g, and 15 min of centrifugation at 10 °C, take the upper organic phase, blow dry under nitrogen, and number for detection. The assay was repeated with 100 μL of isopropanol, and the quality control samples were randomly inserted into the assay sequence of the samples.

### 2.3 Test conditions

#### 2.3.1 Chromatographic conditions

The samples were separated by ultra-high performance liquid chromatography (UHPLC) system. The analysis was performed on a Waters ACQUITY UPLC BEH C18 column (2.1 mm i. d. × 100 mm length, 1.7 μm particle size). The mobile phase consisted of A: 10 mM ammonium formate acetonitrile solution in water and B: 10 mM ammonium formate acetonitrile isopropanol solution. Elution procedure: B was maintained at 30% (0–2 min), B was varied linearly from 30% to 100% (2–25 min), and B was maintained at 30% (25–35 min). The samples were placed in the autosampler at 10 °C during the whole analysis. To avoid the effect caused by the fluctuation of the instrument detection signal, a random order was used and the samples were analyzed continuously.

#### 2.3.2 Mass spectrometry conditions

Electrospray ionization (ESI) positive ion and negative ion modes were used for detection, respectively. Samples were separated by UHPLC and then analyzed by mass spectrometry using a Q Exactive plus mass spectrometer (Thermo Scientific™).

#### 2.3.3 Mass-to-charge ratio (m/z) acquisition of lipid molecules and lipid fragments

The electrospray ionization source operates in positive and negative ion modes respectively. The spray voltage of the MS (primary mass spectrometer) is inferred to be 3.0–4.0 kV in positive mode and 2.5–3.5 kV in negative mode. The temperature of the transfer tube is approximately 300 °C–350 °C, and the full scanning range covers m/z 150-1,500. The resolution reaches 70,000 at m/z 200, the target value of automatic gain control (AGC) is inferred to be 1e6, and the maximum injection time may be 100 m. The MS/MS (secondary mass spectrometry) adopts a data-dependent acquisition mode, with the duty cycle optimized to within 1–2 s. The top 10 parent ions with the highest intensity are selected, and HCD fragmentation (collision energy 20–40 eV) is used. The quadrupole isolation window is m/z 1.0-2.0, and the dynamic exclusion time is set at 10–20 s. The resolution is 17,500 at m/z 200. This method ensures the precise identification and quantification of lipid molecules through high-resolution MS scanning and targeted MS/MS fragmentation.

### 2.4 Data processing

The basal peak profile (BPC) plots of the QC samples were compared for spectral overlap to examine the stability of the instrument and the reproducibility of the experiment. LipidSearch was used for peak identification, peak extraction, and lipid identification. Univariate and multivariate statistical analyses were performed on the extracted data, mainly including t-test, multiple of difference (FC) analysis, principal component analysis (PCA), orthogonal partial least squares discriminant analysis (OPLS-DA) and visualization of volcano and cluster plots. The strength of influence and explanatory power of each lipid molecule on the categorical discrimination of each group of samples was measured by the variable weight value (VIP). Lipid molecules with VIP>1 had significant contribution in the model interpretation. Finally, the metabolic pathway analysis of differential lipid molecules was carried out in combination with the Kyoto Encyclopedia of Genes and Genomes (KEGG) database and Metabo Analyst 5.0 online analysis platform. In this experiment, VIP>1, P < 0.05 and FC > 1.5 or <0.67 were used as criteria to screen for significant differential lipid molecules.

## 3 Results

### 3.1 Characteristics of the general population

There were no statistically significant differences (P > 0.05) between the three groups in terms of age, gender, marital status, education, occupation, history of diabetes, smoking, alcohol consumption, sleep duration and exercise, indicating that the three groups were balanced and comparable Table 1.

**TABLE 1:** Comparison of baseline information for lipidomics study subjects, n (%).

List	Total (n = 51)	NGT (n = 17)	DM (n = 17)	DH (n = 17)	*χ* ^*2*^	*P*
Age					0.605	0.962
<45	5 (9.8)	2 (11.8)	2 (11.8)	1 (5.9)		
45–60	32 (62.7)	10 (58.8)	11 (64.7)	11 (64.7)		
≥60	14 (27.5)	5 (29.4)	4 (23.5)	5 (29.4)		
Gender					0.158	0.924
Male	28 (54.9)	9 (52.9)	10 (58.8)	9 (52.9)		
Female	23 (45.1)	8 (47.1)	7 (41.2)	8 (47.1)		
Marital					1.041	0.594
Married	49 (96.1)	17 (100.0)	16 (94.1)	16 (94.1)		
Other	2 (3.9)	0 (0.0)	1 (5.9)	1 (5.9)		
Educational					7.515	0.111
Primary school	22 (43.1)	4 (23.5)	9 (52.9)	9 (52.9)		
High School	18 (35.3)	8 (47.1)	3 (17.6)	7 (41.2)		
University	11 (21.6)	5 (29.4)	5 (29.4)	1 (5.9)		
Occupation					8.442	0.077
Brain worker	7 (13.7)	4 (23.5)	3 (17.6)	0 (0.0)		
Manual worker	33 (64.7)	12 (70.6)	8 (47.1)	13 (76.5)		
Retiree	11 (21.6)	1 (5.9)	6 (35.3)	4 (23.5)		
Family diabetes					3.4	0.183
Yes	6 (11.8)	0 (0.0)	3 (17.6)	3 (17.6)		
No	45 (88.2)	17 (100.0)	14 (82.4)	14 (82.4)		
Alcohol use					4.577	0.101
Yes	12 (23.5)	3 (17.6)	7 (41.2)	2 (11.8)		
No	39 (76.5)	14 (82.4)	10 (58.8)	15 (88.2)		
Smoker					0.197	0.906
Yes	14 (27.5)	5 (29.4)	4 (23.5)	5 (29.4)		
No	37 (72.5)	12 (70.6)	13 (76.5)	12 (70.6)		
Sleep (h/d)					2.2	0.699
<7	18 (35.3)	6 (35.3)	6 (35.3)	6 (35.3)		
7∼	30 (58.8)	11 (64.7)	10 (58.8)	9 (52.9)		
≥9	3 (5.9)	0 (0.0)	1 (5.9)	2 (11.8)		
Exercise (times/week)					1.417	0.492
<3	27 (52.9)	7 (41.2)	10 (58.8)	10 (58.8)		
≥3	24 (47.1)	10 (58.8)	7 (41.2)	7 (41.2)		

The NGT (Normal Glucose Tolerance) group served as the healthy control population. The DM (Diabetes Mellitus) group comprised patients with diabetes alone, while the DH (Diabetes with Hyperuricemia) group included individuals suffering from both metabolic disorders simultaneously.

### 3.2 Experimental quality control

**FIGURE 1:**
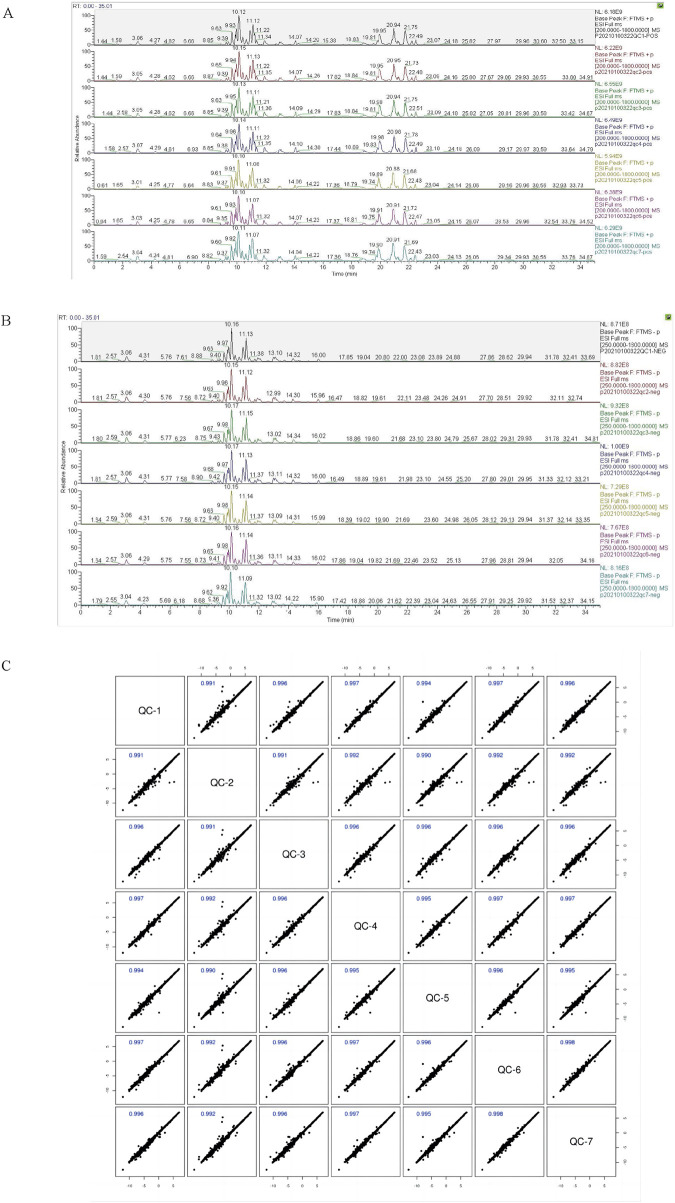
BPC overlay spectra of positive ions **(A)** and negative ions **(B)** from QC samples, and correlation map of QC samples **(C)**. Note: The BPC is the base peak plot; the horizontal coordinates indicate the retention time of each peak, and the vertical coordinates indicate the intensity of the peak; the quality control samples were prepared by mixing equal amounts of case and control samples to be tested. The QC sample correlation map features QC samples on the x-axis and QC samples on the y-axis. Each point within a cell represents an ion peak (metabolite) extracted from the QC sample, with the x- and y-coordinates denoting the logarithmic values of the ion peak signal intensity.

By overlaying the base peak chromatogram (BPC) spectra of quality control (QC) samples and analyzing their correlation maps, distinct separation among different categories of lipid metabolites in plasma was observed. The consistency in peak response intensities and retention times across all QC samples demonstrated high instrument stability, excellent experimental reproducibility, and reliable data quality (Figure 1).

### 3.3 Analysis of lipid components

**FIGURE 2:**
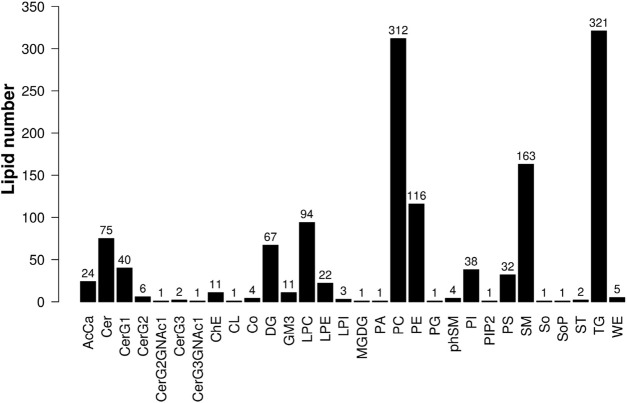
Histogram of lipid subclasses.

Lipid Search software was used to analyze the data, and a total of 30 lipid subclasses and 1,361 lipid molecules were identified in this experiment. There were 621 glycerophospholipids, 388 glycerolipids, 314 sphingolipids, and 38 other lipids (Figure 2).

### 3.4 Multivariate statistical analysis of lipid molecules

#### 3.4.1 Principal component analysis

**FIGURE 3:**
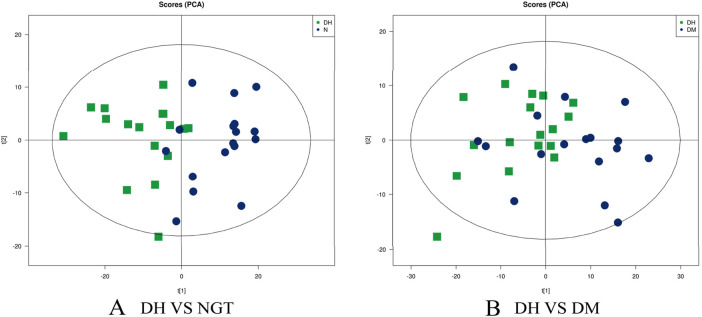
Principal Component Analysis (PCA) Plot. Note: **(A)** shows DH vs. NGT and **(B)** shows DH vs. DM. The horizontal coordinate t (Mozaffarian, 2016) represents principal component 1, the vertical coordinate t (Sun et al., 2022) represents principal component 2, and the ellipse represents the 95% confidence interval. DH, diabetes combined with high uric acid; DM, diabetes mellitus; NGT, normal glucose tolerance.

The PCA method was used to observe the overall distribution trend of samples between groups and the degree of difference of samples between groups. The data were processed to obtain the distribution of metabolites and PCA scores of plasma samples from DH group vs. NGT control group and DM control group, and the results showed that the separation of DH and NGT and DH and DM groups was good. The main parameters of the PCA model model interpretation rate R^2^X = 0.552 (DH vs. NGT) and R^2^X = 0.509 (DH vs. DM), with R^2^X values greater than 0.5 indicating that the model is reliable (Figure 3).

#### 3.4.2 Orthogonal partial least squares discriminant analysis

**FIGURE 4:**
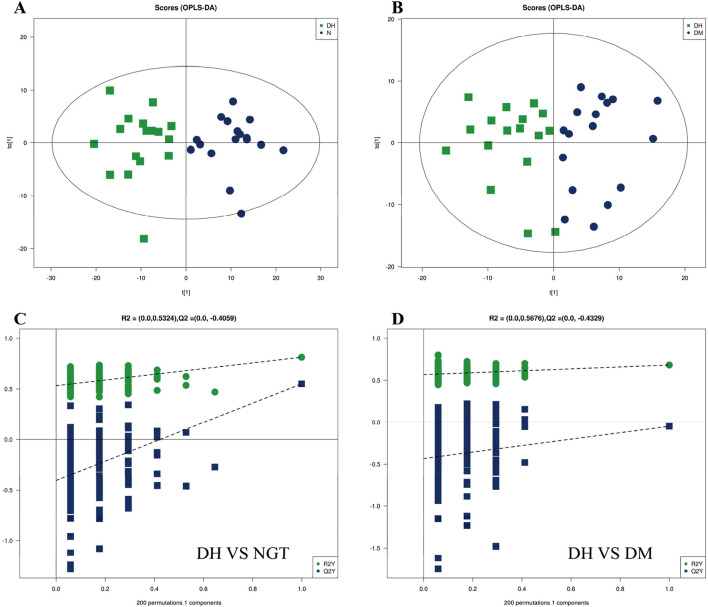
Multivariate analysis of plasma metabolites. Note: **(A)** shows the DH vs. NGT OPLS-DA and **(B)** shows the DH vs. DM OPLS-DA. Figure A/B horizontal coordinate t (Mozaffarian, 2016) represents principal component 1, vertical coordinate t (Sun et al., 2022) represents principal component 2, and the ellipse represents the 95% confidence interval; **(C)** shows the DH vs. NGT replacement results, and **(D)** shows the DH vs. DM replacement results. The horizontal axis of Figure C/D represents the replacement retention, the vertical axis represents the values of *R* ^2^ (green) and Q^2^ (blue), and the two dashed lines represent the regression lines of *R* ^2^ and Q^2^ respectively. OPLS-DA, Orthogonal Partial Least Squares Discriminant Analysis; DH, diabetes combined with high uric acid; DM, diabetes mellitus; NGT, normal glucose tolerance.

The OPLS-DA method was applied to further validate the separation of plasma samples and to screen for differential metabolites associated with the subgroups. The results showed a significant trend of separation between DH and NGT and DH and DM samples, indicating a reliable model. After 7 cycles of cross-validation, the model interpretation rate of R^2^Y = 0.380 and the predictive power Q^2^ = 0.551 were obtained for the DH vs. NGT group, indicating a stable and reliable model (Figure 4A). The model interpretation rate of R^2^Y = 0.682 and the predictive power Q^2^ = 0.167 were obtained for the DH vs. DM group (Figure 4B), indicating a more stable model. The replacement results of both groups showed that the *R* ^2^ and Q^2^ of the stochastic model gradually decreased as the replacement retention gradually decreased, indicating that there was no overfitting in the original model and that the model was robust (Figures 4C, D).

### 3.5 Analysis of plasma lipid molecular differences among the three groups

**FIGURE 5:**
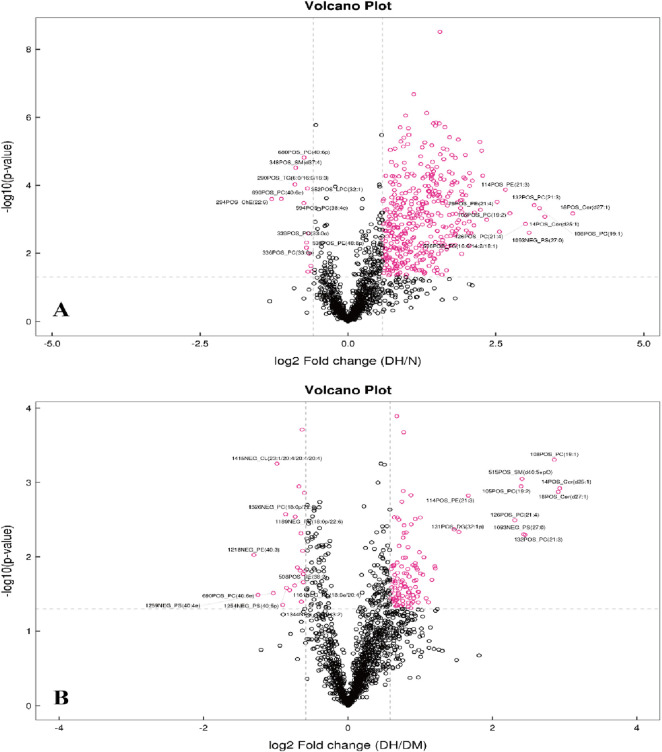
Volcano Plots of Differential Lipid Molecules in DH vs. NGT and DH vs. DM Groups. Note: **(A)** DH vs. NGT; **(B)** DH vs. DM; horizontal coordinates in the figure indicate log2-transformed differential level ploidy values, vertical coordinates indicate log10-transformed *P* value values, and dots indicate lipid molecules, where red dots are lipid molecules that meet the differential level ploidy screening criteria (FC > 1.5 or FC < 0.67, *P* value <0.05). DH, diabetes combined with high uric acid; DM, diabetes mellitus; NGT, normal glucose tolerance; FC fold change; VIP variable importance for the projection.

To analyze the significance of changes in plasma lipid molecules between DH vs. NGT and DH vs. DM groups and to screen for potential lipid biomarkers, this study visualized lipid molecule level by visualizing the overall differential level fold in the comparison groups with FC > 1.5 or FC < 0.67 and P < 0.05 as screening conditions. Compared with the NGT control group, 405 lipid molecules were upregulated and 15 levels were downregulated in the DH group, as shown in Figure 5A. Compared with the DM control group, the DH group exhibited upregulation of 118 lipid molecules and downregulation of 22., as shown in Figure 5B.

### 3.6 Clustering analysis of differential lipid molecules in the three populations

**FIGURE 6:**
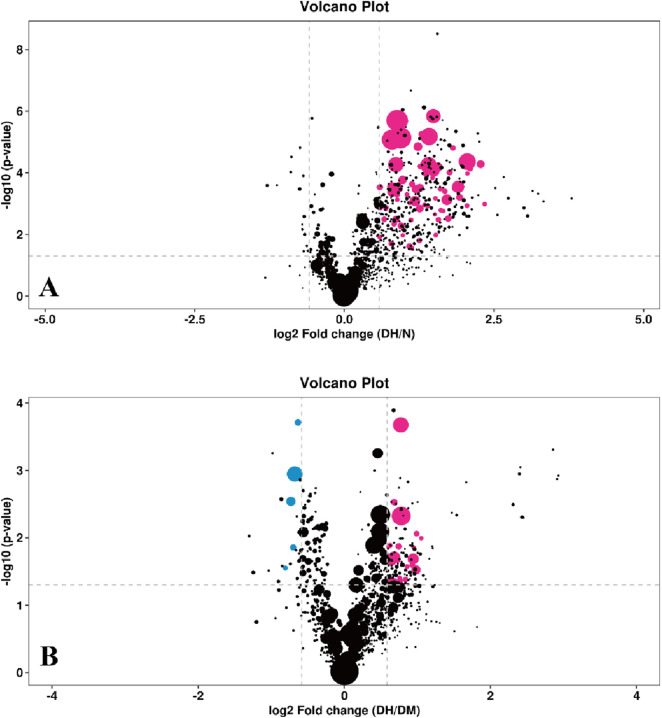
Bubble plot of differential lipid molecules. Note: **(A)** DH vs. NGT; **(B)** DH vs. DM; The horizontal coordinates indicate the log2-transformed difference level multiplier values, the vertical coordinates indicate the log10-transformed P value values, the purple dots indicate lipid molecules that satisfy both FC > 1.5, *P* value <0.05 and VIP >1, the blue dots indicate lipid molecules that satisfy both FC < 0.67, *P* value <0.05 and VIP >1. The larger the air bubble area, the larger the VIP value. DH, diabetes combined with high uric acid; DM, diabetes mellitus; NGT, normal glucose tolerance; FC fold change; VIP variable importance for the projection.

S-plot load plots were used in the OPLS-DA model to obtain the variance variables with high correlation and covariance, and further selected the variables that contributed more to the grouping in the model. The strength of influence and explanatory power of each lipid molecule on the categorical discrimination of each group of samples was measured by the variable weight value (VIP). Lipid molecules with VIP>1 had significant contribution in the model explanation. The information of the three dimensions of differential level multiple, P value and VIP value of lipid molecules were again visualized in the form of bubble plots by combining the FC > 1.5 or FC < 0.67 and P < 0.05 conditions, as shown in Figure 6.

**FIGURE 7:**
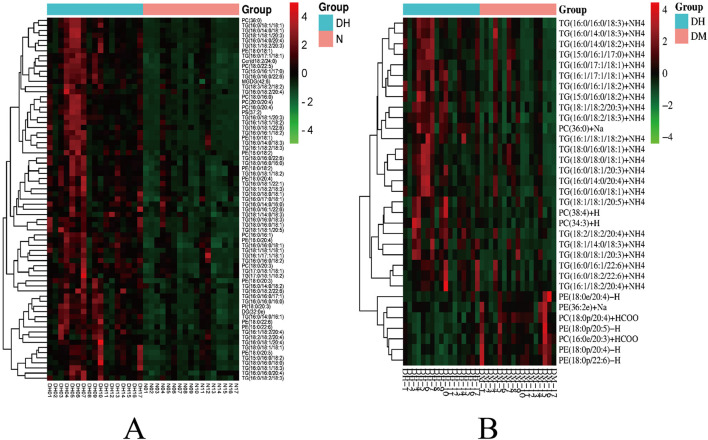
Heatmap of hierarchical clustering analysis for significantly altered plasma lipid molecules. Note: **(A)** DH vs. NGT; **(B)** DH vs. DM; Rows represent lipid molecules, and columns represent individual samples. The color intensity in each cell corresponds to the relative abundance of the lipid molecule (darker shades indicate higher level). Lipid molecules with similar level patterns are clustered together. Key lipid subclasses identified include: TG, triglyceride; PC, phosphatidylcholine; PE, phosphatidylethanolamine; PI, phosphatidylinositol.

FC > 1.5 or FC < 0.67, P < 0.05 and VIP >1 were used to select significantly different lipid molecules between groups and to perform hierarchical clustering of samples from each group, thus assisting us to accurately screen for marker lipids and to investigate changes in related metabolic processes. A total of 73 significantly different lipid molecules were expressed upregulated in the DH group compared with the NGT control group. A total of 34 lipid molecules were significantly altered in the DH group compared to the DM control group, with 27 upregulated level and 7 downregulated level. Figure 7 shows the results of hierarchical clustering of significantly different lipid molecules in DH vs. NGT and the results of hierarchical clustering of significantly different lipid molecules in DH vs. DM. From the figure (Figure 7A), it can be learned that the identified differential lipid metabolites in the DH group compared with the NGT group all had higher level high in the DH group, and the grouping of differential lipid metabolites in both groups was obvious and reproducible. From Figure 7B, it can be learned that the DH group had significantly better reproducibility of differential lipid metabolite grouping compared to the DM group.

Significantly different lipid molecules identified above with FC > 1.5 or FC < 0.67, P < 0.05 and VIP >1 were further designated by MetaboAnalyst 5.0 platform. There were 31 significantly different lipid molecules in the DH group compared to the NGT group, including 13 TGs, followed by 7 phosphatidylcholine (PC), 10 phosphatidylethanolamine (PE) and one phosphatidylinositol (PI) (Table 2). There were 12 significantly different lipid molecules in the DH group compared to the DM group, including 10 TGs and 2 PCs (Table 3).

**TABLE 2:** Significantly altered lipid metabolites in DH compared to NGT plasma.

Lipids	Lipid subclass	Molecular formula	Theoretical mass to charge ratio	Retention period (min)	FC	*P*	VIP
PC(16:0/16:1)+HCOO	PC	C41 H79 O10 N1 P1	776.5447	9.9531	1.8183	3.23 × 10^−3^	2.1227
PC(16:0/20:4)+HCOO	PC	C45 H81 O10 N1 P1	826.5604	9.9588	1.7246	1.98 × 10^−2^	1.5866
PC(18:0/16:0)+Na	PC	C42 H84 O8 N1 P1 Na1	784.5827	10.3784	1.5080	2.92 × 10^−4^	1.7613
PC(18:0/20:3)+HCOO	PC	C47 H87 O10 N1 P1	856.6073	11.3578	1.8167	5.41 × 10^−5^	5.2682
PC(18:0/22:5)+HCOO	PC	C49 H87 O10 N1 P1	880.6073	11.3020	1.7770	4.60 × 10^−3^	1.3706
PC(20:0/20:4)+HCOO	PC	C49 H89 O10 N1 P1	882.6230	11.6216	1.6171	1.45 × 10^−3^	1.3183
PC(36:0)+Na	PC	C44 H88 O8 N1 P1 Na1	812.6140	11.3160	1.7442	4.16 × 10^−4^	3.1812
PE (16:0/18:1)-H	PE	C39 H75 O8 N1 P1	716.5236	11.2232	2.1317	9.34 × 10^−4^	1.1945
PE (16:0/18:2)-H	PE	C39 H73 O8 N1 P1	714.5079	10.4423	1.9056	3.39 × 10^−4^	1.4847
PE (16:0/20:4)-H	PE	C41 H73 O8 N1 P1	738.5079	10.2525	1.8427	5.11 × 10^−4^	1.5114
PE (16:0/22:6)-H	PE	C43 H73 O8 N1 P1	762.5079	9.9431	1.5883	3.20 × 10^−3^	1.5807
PE (18:0/18:1)-H	PE	C41 H79 O8 N1 P1	744.5549	12.2351	2.0671	5.03 × 10^−4^	1.2186
PE (18:0/18:2)-H	PE	C41 H77 O8 N1 P1	742.5392	11.4109	1.9647	1.69 × 10^−4^	2.6506
PE (18:0/20:3)-H	PE	C43 H79 O8 N1 P1	768.5549	11.6504	2.3852	3.56 × 10^−4^	1.0843
PE (18:0/20:4)-H	PE	C43 H77 O8 N1 P1	766.5392	11.2259	1.7332	2.81 × 10^−4^	2.8162
PE (18:0/20:5)-H	PE	C43 H75 O8 N1 P1	764.5236	10.5966	2.2163	2.45 × 10^−4^	1.1841
PE (18:0/22:6)-H	PE	C45 H77 O8 N1 P1	790.5392	10.9251	1.8742	2.47 × 10^−4^	1.6682
PI(18:0/20:3)-H	PI	C47 H84 O13 N0 P1	887.5655	10.3162	1.5062	1.21 × 10^−2^	1.2277
TG (16:0/16:0/18:1)+NH4	TG	C53 H104 O6 N1	850.7858	21.7533	2.6524	5.67 × 10^−5^	5.6638
TG (16:0/16:0/18:2)+NH4	TG	C53 H102 O6 N1	848.7702	20.8156	2.2687	8.21 × 10^−4^	3.3256
TG (16:0/16:0/20:4)+NH4	TG	C55 H102 O6 N1	872.7702	20.6176	3.7993	6.20 × 10^−4^	2.0225
TG (16:0/16:1/18:2)+NH4	TG	C53 H100 O6 N1	846.7545	18.6511	2.7763	1.46 × 10^−4^	1.1419
TG (16:0/18:1/18:1)+NH4	TG	C55 H106 O6 N1	876.8015	21.7168	1.9140	7.30 × 10^−6^	7.6742
TG (16:0/18:1/18:2)+NH4	TG	C55 H104 O6 N1	874.7858	20.9303	1.7354	8.35 × 10^−6^	7.1289
TG (16:0/18:1/20:4)+NH4	TG	C57 H104 O6 N1	898.7858	20.6030	2.8153	8.71 × 10^−5^	3.7601
TG (16:0/18:2/20:4)+NH4	TG	C57 H102 O6 N1	896.7702	19.5268	2.9567	6.51 × 10^−5^	2.9516
TG (16:1/18:1/18:2)+NH4	TG	C55 H102 O6 N1	872.7702	18.9597	2.1633	3.40 × 10^−3^	1.2901
TG (16:1/18:2/20:4)+NH4	TG	C57 H100 O6 N1	894.7545	18.5472	2.8838	1.05 × 10^−2^	1.2103
TG (18:0/18:0/18:1)+NH4	TG	C57 H112 O6 N1	906.8484	23.0595	4.8503	5.22 × 10^−5^	2.4330
TG (18:0/18:1/18:1)+NH4	TG	C57 H110 O6 N1	904.8328	22.4446	2.8051	1.45 × 10^−6^	4.8965
TG (18:2/18:2/20:4)+NH4	TG	C59 H102 O6 N1	920.7702	18.4881	2.5356	1.11 × 10^−3^	1.2670

The number before the ratio in parentheses is the length of the carbon chain, the number after the ratio is the number of double bonds on the carbon chain; three sets of numbers indicate that the compound consists of three longer carbon chains; p indicates a phosphorus-containing group on the carbon chain; +HCOO, +NH4 and -H, are lipid molecule change groups; PC, phosphatidylcholine; PE, phosphatidylethanolamine; PI, phosphatidylinositol; TG, triglyceride; FC, fold change; VIP, variable importance for the projection.

**TABLE 3:** Significantly different lipid metabolites identified in plasma from the DH group compared to the DM group.

Lipids	Lipid subclass	Molecular formula	Theoretical mass to charge ratio	Retention period (min)	FC	*P*	VIP
PC(34:3)+H	PC	C42 H79 O8 N1 P1	7.5655	9.1739	1.5247	1.33 × 10^−2^	1.3857
PC(38:4)+H	PC	C46 H85 O8 N1 P1	8.1060	1.0418	1.5425	1.29 × 10^−2^	1.3319
TG (16:0/14:0/18:2)+NH4	TG	C51 H98 O6 N1	8.2074	1.9748	1.9222	2.06 × 10^−2^	3.5702
TG (16:0/16:0/18:1)+NH4	TG	C53 H104 O6 N1	8.5079	2.1753	1.6046	1.96 × 10^−2^	4.0657
TG (16:0/16:0/18:3)+NH4	TG	C53 H100 O6 N1	8.4675	1.9769	1.7153	4.74 × 10^−3^	6.6316
TG (16:0/16:1/18:2)+NH4	TG	C53 H100 O6 N1	8.4675	1.8651	1.6625	2.17 × 10^−2^	1.2037
TG (16:1/18:1/18:2)+NH4	TG	C55 H102 O6 N1	8.7277	1.8960	1.9847	8.66 × 10^−3^	1.5602
TG (16:1/18:2/20:4)+NH4	TG	C57 H100 O6 N1	8.9475	1.8547	1.9601	5.12 × 10^−2^	1.1011
TG (18:0/16:0/18:1)+NH4	TG	C55 H108 O6 N1	8.7882	2.2469	1.5952	5.39 × 10^−2^	4.3940
TG (18:0/18:0/18:1)+NH4	TG	C57 H112 O6 N1	9.0685	2.3060	1.6926	4.38 × 10^−2^	1.7484
TG (18:1/18:1/20:5)+NH4	TG	C59 H104 O6 N1	9.2279	1.9505	1.5736	2.28 × 10^−2^	2.2372
TG (18:2/18:2/20:4)+NH4	TG	C59 H102 O6 N1	9.2077	1.8488	1.9327	1.39 × 10^−2^	1.0867

The number before the ratio in parentheses is the length of the carbon chain, the number after the ratio is the number of double bonds on the carbon chain, three sets of numbers indicate that the compound consists of three longer carbon chains, p indicates a phosphorus-containing group on the carbon chain; +NH4 and +H are lipid molecule change groups. PC, phosphatidylcholine; PE, phosphatidylethanolamine; PI, phosphatidylinositol; TG, triglyceride; FC, fold change; VIP, variable importance for the projection.

### 3.7 Analysis of plasma differential lipid molecule metabolic pathways

**FIGURE 8:**
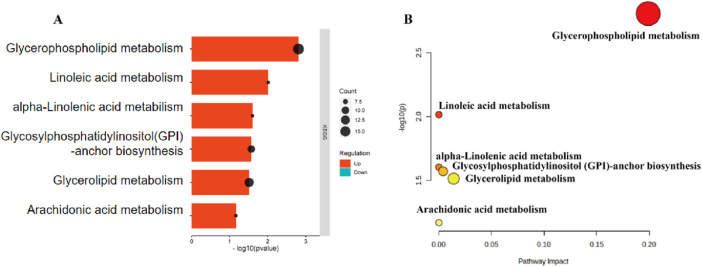
DH vs. NGT plasma significantly different lipid molecule metabolic pathways. Note: **(A)** Bars indicate the size of the P-value; longer bars indicate smaller P-values; dots indicate the number of lipid metabolites; larger dots indicate more lipid metabolites. **(B)** X-axis indicates pathway influence, Y-axis indicates -log(P); significance and statistical significance of pathways are proportional to the radius and colour of the nodes, respectively.

**FIGURE 9:**
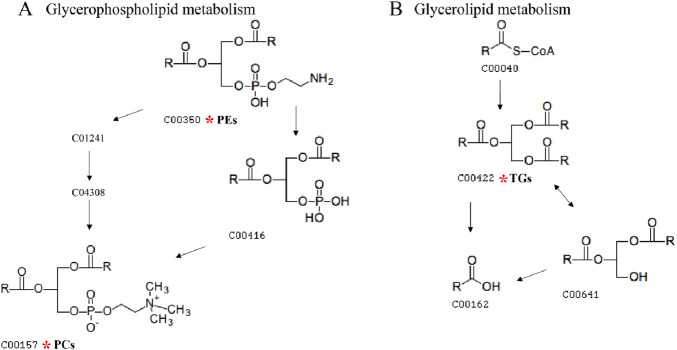
Diagram of glycerophospholipid metabolic pathway and glycerolipid metabolic pathway. Note: **(A)** shows the glycerophospholipid metabolic pathway map, and **(B)** shows the glycerol ester metabolic pathway map. The numbers in the diagrams represent the ID of each lipid subclass; the red * indicates the lipid molecule that is mapped on the KEGG pathway map.

In this study, lipid molecule metabolic pathway analysis was conducted on 31 significantly differential lipid molecules identified by Metabo Analyst 5.0 platform. The results showed that six lipid metabolic pathways were perturbed in DH patients, namely, glycerophospholipid metabolism, linoleic acid metabolism, α-linolenic acid metabolism, glycosylphosphatidylinositol (GPI)-anchor biosynthesis, glycerolipid metabolism, and arachidonic acid metabolic pathway (Figure 8A). Among them, glycerophospholipid metabolic pathway (effect value 0.199) and glycerolipid metabolic pathway (effect value 0.014) were significantly changed (Figure 8B) and were mapped on the KEGG pathway map. Among them, PCs and PEs were mainly involved in glycerophospholipid metabolism (Figure 9A), and triglycerides (TGs) were mainly involved in glycerol ester metabolism (Figure 9B).

**FIGURE 10:**
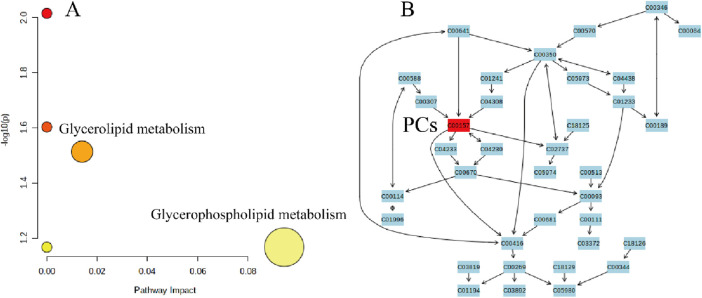
DH vs. DM plasma significantly different lipid molecule metabolic pathways. Note: **(A)** X-axis indicates pathway impact, Y-axis - log(P); pathway significance and statistical significance are proportional to the radius and colour of the node, respectively. **(B)** Numbers in the figure represent the ID of each lipid subclass; red * indicates the lipid molecule mapped on the KEGG pathway map.

KEGG enrichment analysis revealed 12 significantly altered lipid molecules in the DH group compared with DM, which were analyzed for significantly different lipid molecule metabolic pathways. The results showed that the 12 lipid molecules were mainly enriched in two lipid metabolic pathways, namely, glycerophospholipid metabolic pathway (effect value 0.094) and triglyceride metabolic pathway (effect value 0.014) (Figure 10A). Among them, the glycerophospholipid metabolic pathway was significantly changed, and PCs were found to be mainly involved in glycerophospholipid metabolism (Figure 10B).

## 4 Discussion

This study extensively explored the characteristics of lipid metabolites in patients with diabetes mellitus combined with hyperuricemia using non-targeted lipidomics techniques. The results of PCA and OPLS-DA analysis showed a clear trend of separation of the three groups of samples and a reliable model. A total of 73 differential lipid metabolites between diabetic patients, diabetic patients with combined hyperuricemia and healthy controls were identified by combining the conditions of VIP>1, FC > 1.5 or FC < 0.67 and P < 0.05. A total of 31 identifiable differential lipid metabolites were further compared in the Metabo Analyst 5.0 analysis platform. The results of differential lipid metabolic pathway analysis showed that patients with diabetes mellitus combined with high uric acid were significantly associated with glycerophospholipid and glycerolipid metabolic pathways. Among them, PCs and PEs were mainly involved in glycerophospholipid metabolism, and triglycerides (TGs) were mainly involved in glycerolipid metabolism, suggesting that they can be used as potential biomarkers for more in-depth study in patients with diabetes mellitus combined with hyperuricemia.

Glycerophospholipids are the major lipids of mammalian cell membranes and play a crucial role in cellular functions such as signal transduction, regulation of protein functions and transport processes (Ecker and Liebisch, 2014). Among them, phosphatidylcholine (PC) and phosphatidylethanolamine (PE) are the two most dominant phospholipids. In the liver, PC can be synthesized via the choline pathway or converted by PE through the action of phosphatidylethanolamine-N-methyltransferase (PEMT) (Vance, 2015). Phospholipid composition is closely related to hepatic insulin signaling. It was found that an elevated ratio of hepatocyte phosphatidylcholine to phosphatidylethanolamine (PC/PE) can directly affect insulin signaling (Van Der Veen et al., 2019). Conversely, a decrease in PC/PE improves lipid accumulation, inflammation and fibrosis in hepatocyte (Van Der Veen et al., 2016),and facilitates glucose homeostasis in obese mice (Fu et al., 2011). In this study, we found abnormally elevated 7 PCs and 10 PEs in patients with combined diabetes mellitus and hyperuricemia, suggesting abnormal glycerophospholipid metabolism in DH patients. Other studies have also found (Drogan et al., 2015),that alterations in glycerophospholipid classes in patients with type 2 diabetes suggest that PC (22:4/dm18:0), PC (38:4), PC (36:1) and PE (O-18:0/O-18:0) and PE (O-20:0/O-16:0) are positively associated with the risk of diabetes mellitus. In addition, serious disturbances in glycerophospholipid metabolic pathways have been identified in both *in vivo*/*in vitro* studies of hyperuricemia (Liu et al., 2020). It was also found that uric acid induces lipid metabolism disorders through lysophosphatidylcholine acyltransferase 3 (LPCAT3)-mediated activation of sterol regulatory element binding protein 1c (SREBP-1c) and inhibition of phosphorylation signaling transducer and activator of transcription 3 (p-STAT3 (Liu et al., 2020). It is suggested that LPCAT3 may be a key regulator linking hyperuricemia and disorders of lipid metabolism.

Triglycerides (TGs) are the major form of fatty acids stored and transported intracellularly and in the blood plasma. When the body exceeds the maximum storage capacity of adipocytes, lipids are released into the circulation as free fatty acids (FAs) and are transported to accumulate in lipid droplets in skeletal muscle and liver, ultimately leading to insulin resistance (Ritter et al., 2015). However, stored TGs *in vivo* are usually metabolically inert. Impaired insulin signaling in hepatocytes is not caused by the accumulation of triglycerides, but by direct damage to hepatocytes from intermediates produced during their metabolism (e.g., diglycerides, ceramides, and acylcarnitines) (Semova and Biddinger, 2021). In addition, the accumulation of excess lipids (especially saturated fatty acids) is closely associated with endoplasmic reticulum oxidative stress and mitochondrial dysfunction (Wymann and Schneiter, 2008). Also, elevated free fatty acids in plasma can cause a direct inflammatory response by binding to toll-like receptors (SONG et al., 2019). Lipid molecules can also be used as biomarkers of disease. It was found (Yang et al., 2019) that neurolipid dysregulation (especially of TGs) was significantly associated with peripheral neuropathy in a murine model of diabetes and that the disease was associated with the “fat digestion and absorption” pathway through the KEGG website. In addition, elevated serum uric acid is usually accompanied by elevated TG levels. It was found (Liu et al., 2022)that serum levels of all types of TG were higher in patients with hyperuricemia than in normal subjects, which may be related to the activation of phospholipases. The mechanism by which triglycerides (TGs) affect diabetes mellitus combined with hyperuricemia may be related to its role in inflammation (Van Dierendonck et al., 2022), oxidative stress (Sun et al., 2020) and insulin signaling (Smith et al., 2020), which may affect glucose metabolism and insulin resistance.

There are some limitations to this study. First, the relatively small number of participants in each group and the nature of the cross-sectional study methodology itself can limit the interpretation of the findings from a pathophysiological perspective. In addition, because blood samples were collected at multiple different sites, a batch effect may have occurred but could have been mitigated by a standardized sample collection, processing, and storage protocol. Second, other unmeasurable factors, including dietary habits, medications/supplements, and other unknown environmental factors may also have influenced the results of the study; however, we captured a portion of the potential confounders’ in the follow-up analysis to adjust for them. Finally, this study was conducted with a sample of the population in Fujian Province; therefore, the generalizability of our findings to other populations is limited.

## 5 Conclusion

In summary, a total of 31 significantly different lipid metabolites were identified as potential biomarkers of DH by UHPLC-MS/MS technique in this study, and they were enriched in glycerophospholipid metabolism, linoleic acid metabolism, α-linolenic acid metabolism, glycosylphosphatidylinositol (GPI)-anchor biosynthesis, glycerolipid metabolism, and arachidonic acid metabolism pathways. Among them, glycerophospholipid and glycerolipid metabolic pathways are the most important. PCs and PEs are mainly involved in glycerophospholipid metabolic pathway, and TGs are closely related to glycerolipid metabolic pathway.

## Data Availability

The raw data supporting the conclusions of this article will be made available by the authors, without undue reservation.
